# Validation of a novel computerized cognitive function test for the rapid detection of mild cognitive impairment

**DOI:** 10.1186/s12883-022-02997-4

**Published:** 2022-12-07

**Authors:** Minoru Kouzuki, Madoka Miyamoto, Nobuto Tanaka, Katsuya Urakami

**Affiliations:** 1 Department of Biological Regulation, School of Health Science, Faculty of Medicine, Tottori University, 86 Nishi-cho, Yonago, 683-8503 Japan; 2 Department of Dementia Prevention, School of Health Science, Faculty of Medicine, Tottori University, 86 Nishi-cho, Yonago, 683-8503 Japan

**Keywords:** Mild cognitive impairment, Alzheimer’s disease dementia, Computerized cognitive assessment battery, Neuropsychological test

## Abstract

**Background:**

In the present study, we examined the distinguishing ability of a mild cognitive impairment (MCI) assessment tool for rapid screening using a computer (MARC) for Alzheimer’s disease dementia (ADD), MCI, and non-demented controls (NDC) with no cognitive impairment, as well as its validity and reliability, as part of a preliminary trial for the development of the tool.

**Methods:**

A total of 64 participants (23 in the ADD group, 17 in the MCI group, and 24 in the NDC group) were analyzed. The participants were administered MARC and a pre-existing computerized Alzheimer’s dementia screening test (MSP), and 31 participants (14 in the MCI group, 17 in the NDC group) were readministered MARC within 4 months from the first test.

**Results:**

The median (interquartile range) test time for MARC was 401 (350–453) s. Total MARC scores were significantly worse in the MCI and ADD groups than in the NDC group (*p* < 0.05 and *p* < 0.01, respectively). In the receiver operating characteristic (ROC) analysis, the area under the ROC curve (AUC) when comparing the NDC and MCI groups was 0.866 (95% CI, 0.759–0.974), when comparing the NDC and AD groups was 0.989 (95% CI, 0.970–1.000), and when comparing the MCI and AD groups was 0.889 (95% CI, 0.790–0.988). Furthermore, there was a significant correlation with the results of the existing test, MSP (r = 0.839, *p* < 0.001). In addition, the intraclass correlation coefficient (ICC) (1,1) when the first and second MARC scores were compared was 0.740 (95% CI, 0.529–0.865; *p* < 0.001).

**Conclusions:**

MARC is considered capable of distinguishing MCI with high accuracy. The tool has good validity and reliability, and it can be administered in a short period of time without the need for a specialist.

**Supplementary Information:**

The online version contains supplementary material available at 10.1186/s12883-022-02997-4.

## Background

Dementia is thought to develop from a normal state of cognitive function, by going through the stage of mild cognitive impairment (MCI) [1]. There is growing interest in early detection and intervention at the MCI stage. While previous population-based studies have shown that the prevalence of MCI in people aged 65 years and older ranges from a few percent at the lower end to 42% at the higher end, most of the results fall between 5 and 30% [2, 3]. In Japan, an epidemiological survey conducted by the Ministry of Health, Labour and Welfare of Japan reported in March 2013 that the estimated prevalence of MCI was 13% among individuals aged 65 years and over [4]. In addition, the results of a population based prospective cohort study of dementia conducted with approximately 10,000 individuals aged 65 years and above at eight different locations in Japan showed that the prevalence of MCI was 17.0% [5]. Since there are potentially a large number of people with MCI, it is important to establish a system that enables people to have check-ups in their local area and undergo examinations at medical institutions nearby [6, 7] so that they may be evaluated regularly. While the annual conversion rate from MCI to dementia is thought to be approximately 5–15% based on the observation of two review articles [3, 8], MCI does not always exacerbate progressively, and cognitive function may return to normal levels after MCI. It has been shown that, on average, approximately 20% of participants with MCI improve over time [3]. It has been reported that certain active lifestyles may be a contributing factor in the return of cognitive function to normal levels after MCI [9], and the likelihood of cognitive function returning to normal is higher in elderly with MCI who maintain or start multidomain lifestyle activities than those who discontinue or do not engage in such activities [10]. Cognitive function improved in elderly individuals with suspected mild cognitive decline by implementing a program incorporating exercise, cognitive training, and education about dementia and lifestyle habits [11]. These findings suggest that early detection of dementia at the MCI stage and implementation of interventions may reduce the incidence of dementia.

The Montreal Cognitive Assessment (MoCA) was developed as a neuropsychological test for the detection of MCI [12, 13]. It is covered by health insurance in Japan and used as a part of routine clinical practice. MoCA is known for its high sensitivity (90%) and specificity (87%) for detecting MCI in individuals with normal cognitive function, when the cut-off value is set at 25/26 points [12]. The Japanese version of MoCA has also been shown to produce similar results with a sensitivity of 93% and specificity of 89% [13]. However, normative studies using population-based samples showed high false-positive rates by classification using the cut-off value of 26 [14]. Research groups in several countries have conducted normative studies on MoCA, and it has been proposed that the cut-off value should take demographic factors into consideration [14]. The usefulness of MoCA is suggested by the various verifications described above. However, there are difficulties involved in implementing MoCA in local-area check-ups, which requires evaluation of a large number of cases; as MoCA is an individual interview-based test, it is preferable to be administered by a professional with specialized knowledge. On the other hand, while a variety of computerized screening tools have been developed for the detection of MCI, the test time of computerized tests, whose reliability and validity measures were reported in a review by Tsoy et al. [15], was 10 minutes or longer, although they showed high sensitivity and specificity for detecting MCI. In addition, many of the computerized tests shown in other reviews also take more than 10 minutes to complete [16, 17]. Subjects may experience psychological stress and it may be too long to be used for screening at the local-area check-ups. Several computer-based tests that can be performed in a short period of time have also been reported in Japan and other countries [18–21]. Although the accuracy for identifying dementia is sufficient, the accuracy for identifying MCI has not been verified nor is higher than the detection ability for dementia, so there is room for improvement. We thought that this problem could be solved by devising a new computer-based test by taking advantage of the information about tests that have been developed so far.

Therefore, the purpose of the present study was to prepare questions based on previously developed tests for dementia and MCI and perform a preliminary trial for the development of an MCI assessment tool for rapid screening using a computer (MARC), which could be used in local-area check-ups without the need for a specialized examiner and administered in a short period of time with high accuracy. MARC was performed for Alzheimer’s disease dementia (ADD), MCI, and non-demented controls (NDC) with no cognitive impairment to examine its distinguishing ability, to evaluate its validity and reliability, and for its verification.

## Methods

### Patients

A total of 66 outpatients (25 with ADD, 17 MCI, and 24 NDC) aged 65 years or above that visited Shinsei Hospital (Kurayoshi, Japan) between July 2021 and December 2021 were included. All participants were recruited from the hospital’s forgetfulness outpatient department. All participants were diagnosed by experienced neurologists based on medical history, the results of general physical and neurological examinations, laboratory tests, brain imaging tests such as brain computed tomography, magnetic resonance imaging, and/or single photon emission computed tomography, and neuropsychological tests such as the Geriatric Depression Scale 15-item version, the Mini-Mental State Examination, the Alzheimer’s Disease Assessment Scale-Cognitive Subscale, and/or the Touch Panel-type Dementia Assessment Scale [22]. Participants meeting the diagnostic criteria of the National Institute on Aging-Alzheimer’s Association Work groups (NIA-AA) [23] were diagnosed with ADD. However, patients with ADD undergoing treatment with anti-dementia drugs (donepezil, rivastigmine, galantamine, or memantine) were included in the study only if they had reached the maintenance dose for at least 38 weeks. Patients with MCI were those who met the diagnostic criteria of Petersen et al. [24] and whose global score based on clinical dementia rating (CDR) [25] was 0.5. Those who did not meet the diagnostic criteria of NIA-AA or that of Petersen et al. and were diagnosed as having no cognitive impairment were assigned to the NDC group. The exclusion criterion was visual impairment that would present difficulty when performing MARC.

The present study was approved by the ethics committee of Tottori University Faculty of Medicine (No. 20A228). Prior to conducting the research, the participants and those concerned (consent was also obtained from those concerned only if the participants were suffering from ADD or MCI) were informed about the aims of the research and their consent was obtained in writing.

### Procedures

Information including age, sex, years of education, and global CDR score [25] was obtained in interviews or by surveying medical chart data. Following this, the participants of the study were given MARC, as shown below, and an existing computerized test battery for Alzheimer’s disease screening (developed by Nihon Kohden Corporation with the product name “*monowasure soudan proguramu* (MSP)” (which means forgetfulness consultation program)) [18] on the same day. Regarding MARC, to confirm reproducibility, a second trial was performed with participants who visited the hospital less than 4 months after the first MARC and consented to a second test.

### Administration of MARC

We examined the contents of MSP [18], MoCA [12, 13], touch panel-type dementia assessment scale [22], and visuo-spatial memory test [26], and literature on aphasia testing [27] to prepare questions for MARC. MARC comprises nine tasks (immediate recognition task, time orientation task, digit span forward and backward task, visuo-spatial perception task, visual retention task, digit and letter order task, visuo-spatial memory task, object recognition task, and delayed recognition task) with a maximum score of 20 (Table 1). The questions were prepared using Microsoft PowerPoint (PPT) on a computer (Additional file 1: Fig. S1), and the question sentences were recorded. The actual steps of MARC were as follows. The participants were asked to sit in front of a monitor and wear headphones. Next, the questions and choices prepared using PPT were displayed on the monitor, and the pre-recorded question sentences were read out loud to the participants. Because we could not develop a software version of MARC, there was no response when the screen was touched, MARC was administered in a semi-automated manner where the participants were asked to select a choice from the screen by pointing at it, and the examiner recorded their choice on a scoring form. After the participant responded to a question by pointing at a choice, the examiner would immediately press the “next page” button. All the questions were presented to the participants in this manner. The examiner measured the response time using a stopwatch. The test was performed by researchers (MK, MM, or NT) who are also biomedical laboratory scientists. The examiners were not necessarily the same for the first and second examinations. However, in response to the questions from the examinees, the examiners did not explain with any additional information other than what was shown on the computer screen and the explanation of the voice from the headphones.

**Table 1:** Detailed explanation of the computerized cognitive assessment battery

Tasks	Contents	Maximum score	Number of slides
Immediate recognition task	The participant memorizes four words (pine, cow, train, and desk), which are displayed in order at 4 s intervals, and selects the four words they memorized from 12 words.	No points	7
Time orientation task	The participant is asked what day, date, month, and year it is, and selects from the choices.	4	4
Digit span forward and backward task	Counting forward task: five numbers (9, 5, 7, 8, and 3) are read out loud, and the participant selects the numbers in order from a choice of 1 to 9. Counting backward task: three numbers (9, 4, and 1) are read out loud, and the participant selects in reverse order from a choice of 1 to 9.	2	4
Visuo-spatial perception task	A cube is displayed at the top of the screen, and the participant selects, from five choices shown at the bottom of the screen, the choice that matches the cube as seen from a different angle. A question about a triangular prism is then presented in a similar format.	2	2
Visual retention task	A figure consisting of orange, yellow, and blue rectangles overlapping with a white square is displayed on the screen for 5 s, during which the participant memorizes the figure. The participant has to choose the figure they memorized from three choices.	1	3
Digit and letter order task	The participant selects randomly placed numbers (1, 2, 3) and hiragana (a, i, u) in the following order: “1 → a → 2 → i → 3 → u”.	1	2
Visuo-spatial memory task	The participant memorizes a circle with randomly placed numbers (1, 2) and hiragana (a, i), which is displayed for 8 s. After that, the participant selects in the order of “1 → a → 2 → i” from the screen where only a circle is displayed with the numbers and hiragana removed.	1	3
Object recognition task	A picture of a shovel is displayed, and the participant selects its name from six choices.	1	1
Delayed recognition task	The participant selects from 12 choices the four words that they memorized in the immediate recognition task.	8	1

### Implementation of the MSP

MSP is a computerized test battery for ADD screening that was created based on the revised Hasegawa’s Dementia Scale [28]. In the test, the subject performs on their own following the instructions given by the computer (MSP-1100; Nihon Kohden Corporation, Tokyo, Japan) [18]. It comprises four evaluation items: three-word memory test, temporal orientation test, three-dimensional visual-spatial perception test, and delayed recall test. The maximum score is 15 points for answering all questions correctly and the minimum score is 0 points for answering all questions incorrectly. It was reported that the sensitivity and specificity for distinguishing between healthy control and ADD were 96 and 86%, respectively, when the cut-off value was set to 12/13 points [18].

### Statistical analysis

The sample size was based on a previous study that verified the Japanese version of MoCA [13], and was originally defined as 150 patients (50 each in ADD, MCI, and NDC groups), which is slightly larger than that in the previous study. However, because of difficulties in the recruitment process, the sample size was reduced to 66. MARC’s questions were uniquely devised, and we were unable to infer the effect size in the calculation of the sample size due to the lack of similar previous research. Therefore, we did not perform a power analysis in this study.

SPSS version 27 was used for statistical analysis. All data were tested for normality using the Shapiro–Wilk test. The Fisher’s exact test, one-way analysis of variance (ANOVA), or the Kruskal–Wallis test were used to compare demographic characteristics. The Tukey method or Bonferroni method was used for subsequent multiple comparison tests. ANOVA or the Kruskal-Wallis was used to evaluate the results of MARC for the ADD, MCI and NDC groups, and the Tukey method or Bonferroni method was used for subsequent multiple comparison tests. Moreover, the receiver operating characteristic (ROC) curve was plotted and the area under the ROC curve (AUC) and 95% confidence interval (CI) were calculated. In addition, cut-off values, sensitivity, specificity, positive predictive value (PPV), and negative predictive value (NPV) were calculated. Regarding the validity of MARC, the association between MARC and MSP was evaluated by calculating Spearman’s rank correlation coefficient. The internal consistency of MARC was evaluated by calculating the coefficient of Cronbach’s alpha. The reproducibility of MARC was evaluated by calculating the intraclass correlation coefficient (ICC (1, 1)) and 95% CI using the results of those who were able to perform the MARC twice. All statistics were two-tailed and *p* < 0.05 was considered significant.

## Results

Participant data are shown in Table 2. In the present study, two patients with ADD who were unable to complete MARC were excluded from the 66 participants enrolled, and 64 participants were analyzed (23 participants in the ADD group, 17 participants in the MCI group, and 24 participants in the NDC group). Age was significantly higher, and years of education was significantly lower in the ADD group than in the NDC group (both *p* < 0.01). With regard to CDR, all participants in the NDC group had no dementia with a CDR of 0, all participants in the MCI group had questionable dementia with a CDR of 0.5, and in the ADD group, five participants had mild dementia with a CDR of 1 and 18 participants had moderate dementia with a CDR of 2.

**Table 2:** Characteristics of patients

	NDC	MCI	ADD	*P*-value
	(*n* = 24)	(*n* = 17)	(*n* = 23)	
Sex (M:F)	7:17	9:8	8:15	0.302
Age (years)	79.5 (75.8–82.0)	82.0 (79.0–85.0)	85.0 (80.0–88.5)	< 0.001 ^a^
Education (years)	12.0 (11.5–13.3)	12.0 (9.0–12.0)	9.0 (9.0–12.0)	0.008 ^a^
CDR staging (0/0.5/1/2)	24/0/0/0	0/17/0/0	0/0/5/18	–

Data are presented as numbers or medians (interquartile range)

Sex was analyzed using the Fisher’s exact test. Age was compared using a one-way analysis of variance followed by the Tukey test. Education was compared using the Kruskal–Wallis test followed by the Bonferroni correction

*CDR* clinical dementia rating, *NDC* non-demented controls, *ADD* Alzheimer’s disease dementia, *MCI* mild cognitive impairment

^a^ Significant difference between ADD and NDC (*p* < 0.01)

**Fig. 1:**
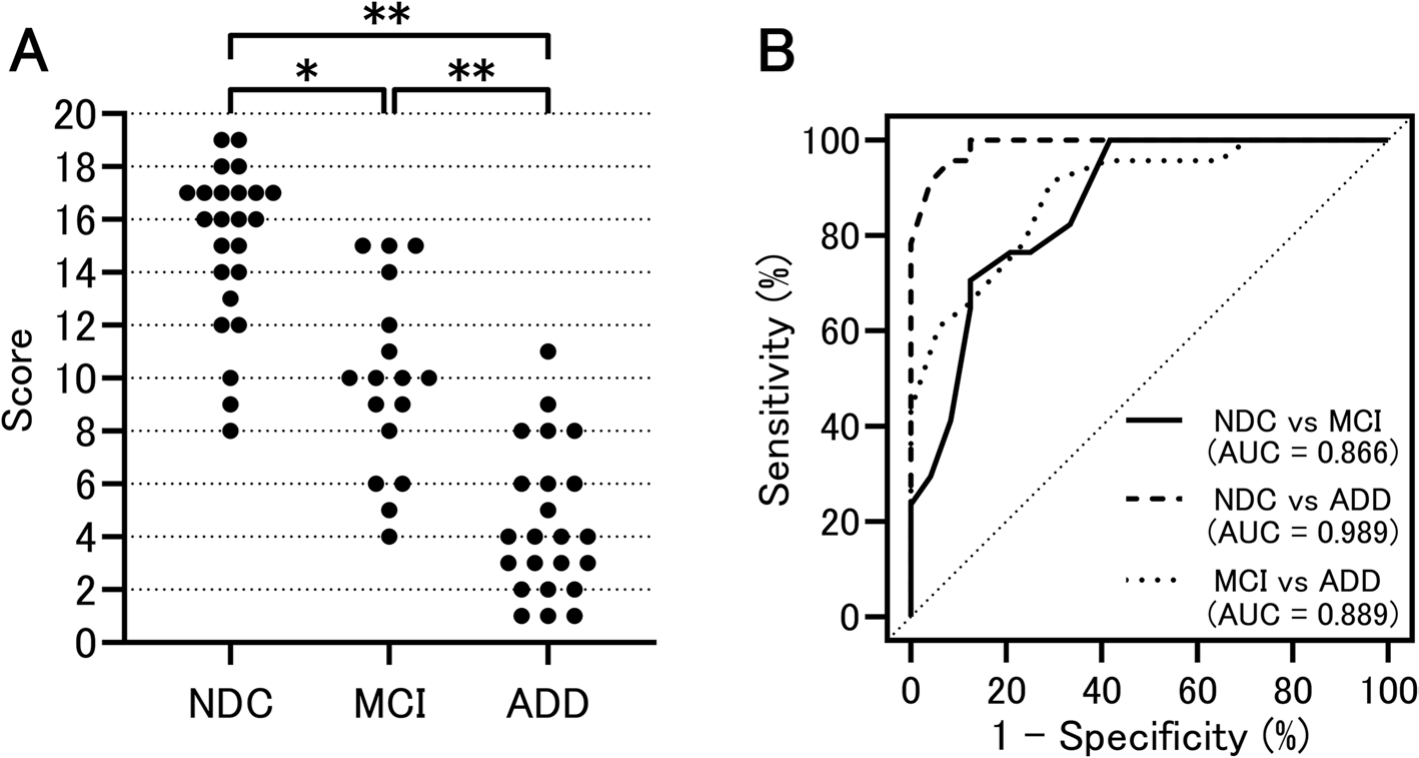
Diagnostic performance of the MARC. **A** The score of MARC in patients with ADD, MCI, and NDC. The MCI and ADD groups scored significantly worse than the NDC group (*p* < 0.05 and *p* < 0.01, respectively), and the ADD group scored significantly worse than the MCI group (*p* < 0.01). **B** ROC analysis in patients with ADD, MCI, and NDC. The AUC was 0.866 for NDC versus the MCI group, 0.989 for NDC versus the ADD group, and 0.889 for MCI versus the ADD group. The data was compared using the Kruskal–Wallis test, followed by the Bonferroni correction. * *p* < 0.05, ** *p* < 0.01. NDC, non-demented controls; ADD, Alzheimer’s disease dementia; MCI, mild cognitive impairment; MARC, mild cognitive impairment assessment tool for rapid screening using a computer; ROC, receiver operating characteristic; AUC, area under the receiver operating characteristic curve

Figure 1A shows a comparison of MARC scores among the three groups. The MCI group and the ADD group scored significantly worse than the NDC group (*p* < 0.05 and *p* < 0.01, respectively), and the ADD group scored significantly worse than the MCI group (*p* < 0.01). The median time (interquartile range) that the participants required to complete the test was 401 (350–453) s. The breakdown by group was 371 (304–405) s for the NDC group, 403 (355–479) s for the MCI group, and 443 (385–511) s for the ADD group. The examination time was significantly longer (*p* < 0.01) in the ADD group than the NDC group. In an ROC analysis conducted to examine the extent to which MARC distinguished the NDC group from the MCI or ADD group, the AUC in the comparison between the NDC and MCI groups was 0.866 (95% CI, 0.759–0.974), that in the comparison between the NDC and ADD groups was 0.989 (95% CI, 0.970–1.000), and in the comparison between the MCI and ADD groups was 0.889 (95% CI, 0.790–0.988) (Fig. 1B). Table 3 shows the sensitivity, specificity, PPV, and NPV for each cut-off value. The optimal cut-off value as suggested by the Youden index was 11/12 points for distinguishing both the MCI group from the NDC group and the ADD group from the NDC group. Further, the participants were stratified by age into groups of 65–74 years, 75–84 years, and 85 years or above (Additional file 1: Table S1), in order consider the effects of age. The analysis results are shown in Additional file 1: Fig. S2. Because the number of participants between the ages of 65 and 74 years and those aged 85 years or above was small, we did not conduct a comparison of the three groups by statistical analysis. However, as with the examination of all participants, examination of the participants between the ages of 75–84 showed that the MCI and ADD groups scored significantly worse than the NDC group (*p* < 0.01 and *p* < 0.01, respectively), while the ADD group scored significantly worse than the MCI group (*p* < 0.01). In addition, the comparison results among the three groups of the MARC subtest are shown in Additional file 1: Table S2. Items in which the MCI group scored significantly lower compared to the NDC group were time orientation task, visuo-spatial memory task, delayed recognition task (all *p* < 0.05), and the ADD group scored lower than the NDC group in the time orientation task, digit span forward and backward task, visuo-spatial perception task, digit and letter order task, and delayed recognition task (all *p* < 0.05).

**Table 3:** Results of sensitivity, specificity, PPV, and NPV in distinguishing MCI and ADD from NDS

Cut-off value	NDC vs MCI				NDC vs ADD			
	Sen	Spe	PPV	NPV	Sen	Spe	PPV	NPV
7 / 8	23.5	100	100	64.9	78.3	100	100	82.8
8 / 9	29.4	95.8	83.3	65.7	91.3	95.8	95.5	92.0
9 / 10	41.2	91.7	77.8	68.8	95.7	91.7	91.7	95.7
10 / 11	64.7	87.5	78.6	77.8	95.7	87.5	88.0	95.5
11 / 12	70.6	87.5	80.0	80.8	100	87.5	88.5	100
12 / 13	76.5	79.2	72.2	82.6	100	79.2	82.1	100
13 / 14	76.5	75.0	68.4	81.8	100	75.0	79.3	100
14 / 15	82.4	66.7	63.6	84.2	100	66.7	74.2	100
15 / 16	100	58.3	63.0	100	100	58.3	69.7	100

Data are presented as percentages (%)

*NDC* non-demented controls, *ADD* Alzheimer’s disease dementia, *MCI* mild cognitive impairment, *Sen* sensitivity, *Spe* specificity, *PPV* positive predictive value, *NPV* negative predictive value

**Fig. 2:**
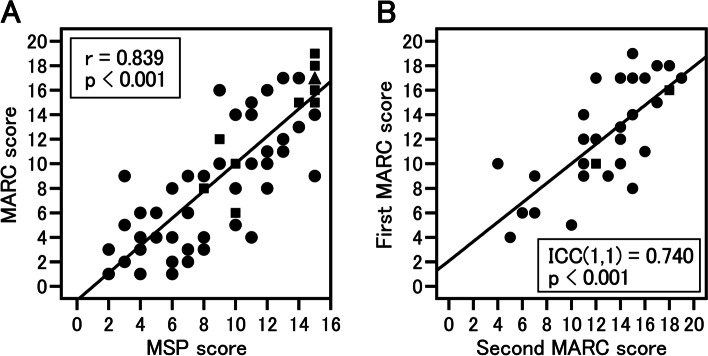
Assessing the validity and reliability of MARC. **A** Scatter plot for the scores in MARC and MSP, an existing computerized cognitive test battery. There was a significant correlation between MARC and MSP (r = 0.839, *p* < 0.001). (B) Scatter plot of the first and second MARC scores with a median 77-day interval. The ICC between the first and second test was 0.740 (*p* < 0.001). A circle indicates 1 person, square indicates 2 people, and triangle refers to 4 people. Correlation analyses were conducted using Spearman’s correlation coefficient. To assess test–retest reliability, ICC were calculated between scores at the first and second tests. MARC, mild cognitive impairment assessment tool for rapid screening using a computer; ICC, intraclass correlation coefficient; MSP, a computerized test battery for Alzheimer’s disease screening (produced by Nihon Kohden Corporation, named “*monowasure soudan proguramu*” (forgetfulness consultation program))

We conducted a correlation analysis with an existing test, MSP, to examine the validity, and a significant correlation was observed (r = 0.839, *p* < 0.001) (Fig. 2A). The Cronbach’s alpha coefficient calculated to examine internal consistency was 0.618. MARC was performed twice with 31 participants (14 in the MCI group and 17 in the NDC group) to study the reproducibility (median time elapsed before re-examination (interquartile range) was 77 (77–77) days). When the first and second MARC scores were compared, the ICC (1,1) was 0.740 (95% CI, 0.529–0.865; *p* < 0.001) (Fig. 2B).

## Discussion

MARC was shown to be a valid evaluation method for cognitive function as it demonstrated a correlation with an existing test, MSP. The reliability was also substantial with an ICC of 0.740 for the first and second tests. Internal consistency was somewhat low with a Cronbach’s alpha coefficient of 0.618, but this is thought to be because various cognitive functions including memory, visuospatial cognition, attention, and executive function were measured. Although MARC could be performed in most of the subjects, two patients with ADD were unable to complete MARC. This suggests that this test may not be possible for people with advanced cognitive impairment to complete. Additionally, it has been shown that the rate of internet usage decreases with age [29], and there is a possibility that many elderly people are unfamiliar with computers. Other cognitive assessments using mobile devices have also shown that there were cases where the test could not be completed in older adult population [30]. Careful instructions on how to operate the machine are necessary when conducting computerized test.

When the MARC cut-off value was set at 11/12, MARC was able to accurately distinguish between the NDC and ADD groups with a sensitivity and specificity of 100 and 87.5%, respectively. However, it should be noted that approximately 78% of the participants in the ADD group had a CDR of 2, indicating moderate ADD. If most of the participants in the ADD group had a CDR of 1, the distinguishing ability of the MARC between NCD and ADD as well as between ADD and MCI is probably lower. Therefore, care should be taken when analyzing the results. On the other hand, the sensitivity and the specificity were 70.6 and 87.5%, respectively, when the cut-off value for distinguishing the MCI group from the NDC group was set at 11/12. The sensitivity was somewhat low because a few patients in the MCI group had high scores. The present study provides the results for each cut-off value (Table 3). We recommend the selection of a cut-off value with high sensitivity according to purpose when using MARC as a screening test for cognitive function impairment in local area check-ups. In addition, the MARC total scores between the MCI and ADD groups were significantly different. However, many patients from both groups scored between 4 and 11 points. Since MARC is a highly difficult test created for the purpose of detecting MCI, low scores of 4–11 points were seen in the MCI group. The overall median test time was 401 s, with the ADD group requiring the longest test time. However, most of the participants finished the test within 10 min. Several computerized cognitive tests validated for MCI, as reviewed by Tsoy et al. [15], have test time of 12–30 minutes, sensitivity of 41–90%, specificity of 64–94%, and the test with the highest sensitivity and specificity required more than 20 minutes. On the other hand, in recent years, there have been several reports of computer-based examinations that have been verified for MCI in Japan as well [19, 31–33]. Although many of these tests have shorter test times than MARC, the specificity and AUC of MARC were the highest (Additional file 1: Table S3). From the above evidence, tests with a higher distinguishing ability for diseases than MARC are thought to take longer for testing, while tests with a shorter test time than MARC are thought to have a slightly inferior distinguishing ability than MARC. As such, MARC is moderate in terms of its distinguishing ability and test time, which positions it in the middle among other tests. In recent years, an increasing number of studies have been utilizing mobile devices for the cognitive assessment of the elderly [30], and there is a growing interest. In view of studies conducted worldwide on computerized cognitive function tests, it is desirable to examine the use of a test based on test time and diagnostic performance. We believe that 10 minutes is acceptable in screening, and MARC is considered to have high performance in distinguishing MCI given that it can be completed within 10 minutes. Currently, a software version of the MARC was developed based on the verification results of the present study. It is a test that can be performed by anyone who owns a computer and does not require the purchase of special equipment. In addition to the advantage of being easy to introduce in terms of cost, it can reduce the burden on the examiner.

The costs and social support associated with dementia increase as the severity of it increases [34, 35]. Additionally, it has been reported that early symptomatic treatment of dementia is cost-effective [36]. In other words, early detection and implementation of appropriate measures are not only important for reducing the burden of care and social costs, but are also beneficial for the patients themselves. However, it has been reported that mild anosognosia is observed in MCI and severe anosognosia in dementia [37]. In other words, subjective evaluation alone (for example, self-report) may not be sufficient as a means for early detection and, therefore, objective evaluation is important. In a study involving a questionnaire survey with elderly individuals, more than half of the respondents were unwilling or undecided regarding regular dementia screening tests. The primary reasons were “bothersome to visit the clinic” and “do not know which doctors can be consulted” [38]. Because there are people who feel resistance towards hospital visits, we believe that, in addition to recommending hospital visits, it is effective for early detection to provide a venue in local areas where people can casually visit to receive a check-up. Furthermore, past studies have shown that participants in home-visit surveys were more likely to experience cognitive decline than those in venue surveys [39], suggesting the importance of home-visit surveys. Thus, approaches including community check-ups and home-visit surveys are necessary for the early detection of cognitive function decline, and a tool that enables evaluation in a short period of time without the need for a specialist is desirable in such settings. We, therefore, believe that the use of computerized tests such as MARC provides high value. In addition, research is ongoing to examine whether preclinical AD can be identified and risk of cognitive decline and ADD can be predicted by administering a new online test, TAS Test, that assesses a range of motor-cognitive functions using a laptop or desktop computer with a webcam and microphone [40], and it is expected that patients in the pre-MCI stage may be detected with the use of computers in the future.

Because MARC is a test prepared in Japanese, it cannot be used with people who do not understand Japanese. However, we believe the questions can be directly translated without any problems, since the MARC questions do not include any cultural questions (that is, questions that only the Japanese people can understand). It should be noted that the words used in the delayed recognition task were chosen from words which the Japanese elderly recalled frequently as presented in a previous study [41], within the categories of animals, vehicles, plants, and furniture. Directly translating these words will result in changes in familiarity with them due to cultural differences. It has been shown that memory performance varies between familiar words and unfamiliar words [42], and this may alter the difficulty of a question. Therefore, words which can be easily recalled by the elderly in each country should be selected from four different categories without changing the concept of question preparation.

Finally, we discuss some of the limitations of the present study. First, the sample size was small. The number of patients in the MCI group was smaller than those in the NDC and ADD groups. In addition, the number of participants between the age of 65 and 74 years was small. Measures for patients during this chronological period are very important to prevent the development of dementia. Furthermore, this is a single-centre study; a multicentre joint study with a larger number of cases which also includes participants between 65 and 74 age is necessary to draw a conclusion. Second, a software version of the test was not available. Although the examiners in this study were specialists, they did not provide more than the necessary explanations, so it is unlikely that the presence of the examiners affected the results. However, non-software version of the test resulted in the participants not receiving any response after selecting an answer on the screen. It is possible that this bewildered the participants and affected their performance. We speculate that those who scored particularly low in the NDC group may have been affected by this. This is a weakness of the study. A software version of the test was developed based on the verification results of the present study. We believe it is important to check the consistency of the results by re-examination. Third, only participants with ADD were tested in the present study and patients with other types of dementia were not evaluated. It is unclear whether other types dementia can be detected as was possible with ADD because the cognitive areas affected vary by the type of dementia. Examination of MCI, which is broadly divided into the amnestic type with memory impairment and non-amnestic type without memory impairment [1], by subtype was also not performed. Although MARC includes questions that assess cognitive functions other than memory, there are many memory-focused questions. Therefore, this test may potentially fail to classify non-amnestic patients. A previous study using MoCA showed that the classification accuracy for non-amnestic MCI was lower than that for amnestic MCI, and there was a difference in the detection rate of MCI subtypes [43]. We believe that an accuracy application of MARC can be found by examining dementia other than ADD and exploring MCI by subtype.

## Conclusions

MARC is a computer-assisted test which can be performed in less than 10 minutes without the need for a specialist. It has high validity in relation to existing tests and high reliability, as evaluated by retesting. This evaluation method is characterized by its ability to distinguish MCI with high accuracy. We believe that MARC can be used as a tool for the early detection of cognitive decline during community check-ups, where many individuals need to be evaluated within a short period of time, as well as home-visit surveys. In addition, computer-based examinations, not person-to-person examinations, are useful in countermeasures against infectious diseases caused by droplet infection, such as the coronavirus disease 2019. In this study, verification was performed by a semi-automated method, but since the software version of MARC has already been developed, we believe that use of the software version of MARC can make the most of the above advantages.

## Supplementary Information

**Additional file 1: Table S1.** Characteristics of patients in the 65–74, 75–84, and over 85 years old groups. **Table S2.** Comparison of the score of subtests in the MARC. **Table S3.** Overview of the psychometric properties of Japanese computerized cognitive function test verified for MCI. **Figure S1.** Samples of the representative tests on the MARC. **Figure S2.** The score of MARC in the 65–74, 75–84, and over 85 years old groups.

## Data Availability

The datasets used and/or analyzed during the current study are available from the corresponding author on reasonable request, subject to the approval of all authors and the ethics committee. Additionally, a software version of MARC (in Japanese) is available free of charge from the corresponding author on reasonable request.

## Abbreviations

ADD: Alzheimer’s disease dementia
MCI: mild cognitive impairment
NDC: non-demented controls
MoCA: Montreal cognitive assessment
MARC: mild cognitive impairment assessment tool for rapid screening using a computer
CDR: clinical dementia rating
ROC: receiver operating characteristic
AUC: area under the receiver operating characteristic curve
CI: confidence interval
ICC: intraclass correlation coefficients
ANOVA: analysis of variance
MSP: a computerized test battery for Alzheimer’s disease screening (produced by Nihon Kohden Corporation, named “*monowasure soudan proguramu*” (forgetfulness consultation program))
