# Supplementary material for: Validation of a novel computerized cognitive function test for the rapid detection of mild cognitive impairment
Source: BMC Neurol. 2022 Dec 7;22:457. doi: 10.1186/s12883-022-02997-4 (PMC9727980; doi:10.1186/s12883-022-02997-4)
Supplement: Supplementary file 1 — Additional file 1: Table S1. Characteristics of patients in the 65–74, 75–84, and over 85 years old groups. Table S2. Comparison of the score of subtests in the MARC. Table S3. Overview of the psychometric properties of Japanese computerized cognitive function test verified for MCI. Figure S1. Samples of the representative tests on the MARC. Figure S2. The score of MARC in the 65–74, 75–84, and over 85 years old groups. [file 12883_2022_2997_MOESM1_ESM.docx]

**Additional file 1**

Table S1. Characteristics of patients in the 65-74, 75-84, and over 85 years old groups.

|  | NDC |  | MCI |  | ADD |  | P-value |
| --- | --- | --- | --- | --- | --- | --- | --- |
| 65 - 74 years old |  |  |  |  |  |  |  |
| Number | 5 |  | 0 |  | 1 |  |  |
| Sex (M:F) | 2:3 |  | - |  | 0:1 |  | - ^a^ |
| Age (years) | 71.0 (70.0 - 71.0) |  | - |  | 72.0 |  | - ^a^ |
| Education (years) | 14.0 (12.0 - 14.0) |  | - |  | 9.0 |  | - ^a^ |
| CDR staging (0/0.5/1/2) | 5/0/0/0 |  |  |  | 0/0/1/0 |  | - |
| 75 - 84 years old |  |  |  |  |  |  |  |
| Number | 17 |  | 12 |  | 9 |  |  |
| Sex (M:F) | 4:13 |  | 6:6 |  | 3:6 |  | 0.381 |
| Age (years) | 81.0 (78.0 - 82.0) |  | 80.5 (78.5 - 82.3) |  | 80.0 (79.0 - 82.0) |  | 0.923 |
| Education (years) | 12.0 (12.0 - 12.0) |  | 12.0 (9.0 - 12.0) |  | 10.0 (9.0 - 12.0) |  | 0.159 |
| CDR staging (0/0.5/1/2) | 17/0/0/0 |  | 0/12/0/0 |  | 0/0/2/7 |  | - |
| Over 85 years old |  |  |  |  |  |  |  |
| Number | 2 |  | 5 |  | 13 |  |  |
| Sex (M:F) | 1:1 |  | 3:2 |  | 5:8 |  | - ^a^ |
| Age (years) | 87.5 (86.3 - 88.8) |  | 86.0 (86.0 - 87.0) |  | 88.0 (87.0 - 91.0) |  | - ^a^ |
| Education (years) | 8.0 (7.0 - 9.0) |  | 11.0 (10.0 - 12.0) |  | 9.0 (9.0 - 12.0) |  | - ^a^ |
| CDR staging (0/0.5/1/2) | 2/0/0/0 |  | 0/5/0/0 |  | 0/0/2/11 |  | - |

Data are presented as number or median (interquartile range).

In the 75-84 years old group, sex was analyzed using the Fisher's exact test, age was compared using a one-way analysis of variance followed by the Tukey test, and education was compared using the Kruskal–Wallis test followed by the Bonferroni correction.

CDR, clinical dementia rating; ADD, Alzheimer's disease dementia; MCI, mild cognitive impairment; NDC, non-demented controls.

^a^ Due to the small number of participants, no comparison using statistical analysis was made between the three groups.

Table S2. Comparison of subtests scores in the MARC.

|  |  | NDC |  | MCI |  | ADD |  | P value |
| --- | --- | --- | --- | --- | --- | --- | --- | --- |
|  |  | (n = 24) |  | (n = 17) |  | (n = 23) |  |  |
| Immediate recognition task |  | - ^a^ |  | - ^a^ |  | - ^a^ |  |  |
| Time orientation task |  | 4 (4 - 4) |  | 2 (0 - 4) |  | 0 (0 - 1) |  | <0.001 ^b, c, d^ |
| Digit span forward and backward task |  | 1.5 (1 - 2) |  | 1 (1 - 1) |  | 0 (0 - 1) |  | <0.001 ^b, d^ |
| Visuo-spatial perception task |  | 2 (1 - 2) |  | 1 (1 - 2) |  | 1 (1 - 1) |  | 0.003 ^b^ |
| Visual retention task |  | 0 (0 - 1) |  | 0 (0 - 1) |  | 0 (0 - 0) |  | 0.153 |
| The digit and letter order task |  | 0 (0 - 1) |  | 0 (0 - 0) |  | 0 (0 - 0) |  | 0.003 ^b^ |
| Visuo-spatial memory task |  | 0 (0 - 0.3) |  | 0 (0 - 0) |  | 0 (0 - 0) |  | 0.020 ^c^ |
| Object recognition task |  | 1 (1 - 1) |  | 1 (1 - 1) |  | 1 (1 - 1) |  | 1.000 |
| Delayed recognition task |  | 6 (6 - 8) |  | 6 (2 - 6) |  | 0 (0 - 3) |  | <0.001 ^b, c, d^ |

Data presented as median (interquartile range).

All items were compared using the Kruskal–Wallis test followed by the Bonferroni correction.

^a^ Results were not shown because no points were assigned.

^b^ Significant difference between ADD and NDC (p<0.05).

^c^ Significant difference between MCI and NDC (p<0.05).

^d^ Significant difference between ADD and MCI (p<0.05).

NDC, non-demented controls; MCI, mild cognitive impairment; ADD, Alzheimer's disease dementia; MARC, mild cognitive impairment assessment tool for rapid screening using a computer.

Table S3. Overview of the psychometric properties of Japanese computerized cognitive function test verified for MCI.

| Source publication | Test | Time | Control vs MCI | | | Correlation  coefficients | Internal consistency | Test-retest reliability |
| --- | --- | --- | --- | --- | --- | --- | --- | --- |
|  |  |  | Sen | Spe | AUC |  |  |  |
| Our study | MARC | median 6.7 (IQR: 5.8 - 7.6) min | 70.6% | 87.5% | 0.866 | MSP: 0.839 | 0.618 | 0.740 |
| Noguchi-Shinohar M, et al (2020) [19] | C-ABC | approximately 5 min | 77.0% | 64.0% | 0.741 | MMSE: 0.753 | NR | NR |
| Takechi H, et al (2021) [31] | CogEvo | approximately 10 min | 81.8% | 57.9% | 0.747 | MMSE: 0.616 | NR | 0.892 |
| Fukui Y, et al (2015) [32] | Touch-panel screening test  (Flipping cards game) | Control: mean 19.3 ± 9.5 sec MCI: mean 30.9 ± 18.4 sec AD: mean 44.0 ± 28.9 sec | 76.9% | 70.7% | NR | AS: 0.234  ABS: 0.142 (ns) | NR | NR |
| Oyama A, et al (2019) [33] | Rapid cognitive assessment using eye-tracking technology | approximately 3 min | NR | NR | 0.845 | MMSE: 0.740 ADAS: -0.640  FAB: 0.570 | NR | NR |

MARC, mild cognitive impairment assessment tool for rapid screening using a computer; C-ABC, computerized assessment battery for cognition; NR, not reported; ns, not significant; MCI, mild cognitive impairment; AD, Alzheimer's disease; MSP, MSP, a computerized test battery for Alzheimer’s disease screening (produced by Nihon Kohden Corporation, named “monowasure soudan proguramu” (forgetfulness consultation program); MMSE, Mini-Mental State Examination; AS, apathy scale; ABS, Abe’s behavioral and psychological symptoms of dementia; ADAS, Alzheimer’s Disease Assessment Scale-cognitive subscale; FAB, Frontal Assessment Battery; Sen, sensitivity; Spe, specificity; AUC, area under the receiver operating characteristic curve.


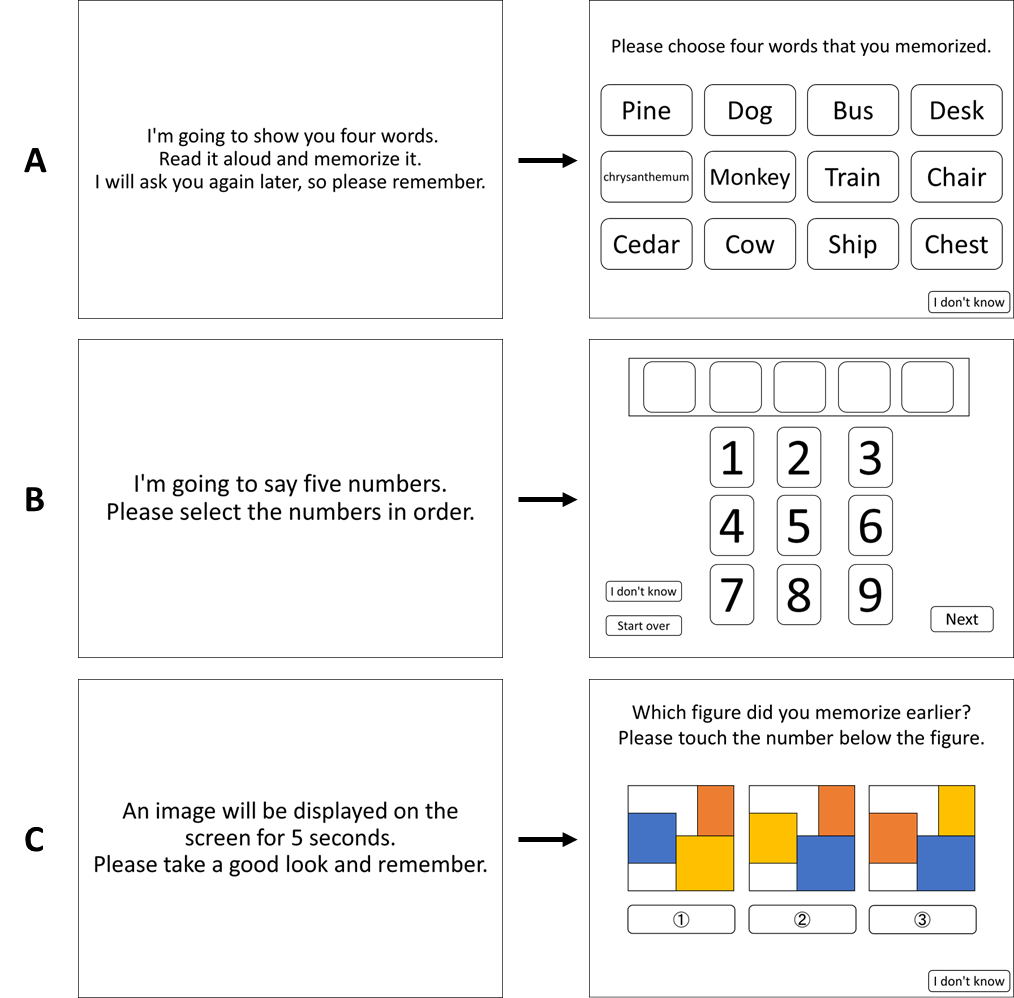


Fig. S1 Samples of the representative tests on the MARC (The texts are translated from Japanese).

(A) Immediate recognition task, the question " I'm going to show you four words. Read it aloud and memorize it. I will ask you again later, so please remember." has been presented (left slide), and the participant selects the four words they memorized from 12 words (right slide). (B) Digit span forward task, the question " I'm going to say five numbers. Please select the numbers in order." has been presented (left slide), and the participant selects the numbers in order from a choice of 1 to 9 (right slide). (C) Visual retention task, the question " An image will be displayed on the screen for 5 seconds. Please take a good look and remember." has been presented (left slide), and the participant has to choose the figure they memorized from three choices.

MARC, mild cognitive impairment assessment tool for rapid screening using a computer.

Fig. S2 The score of MARC in patients with ADD, MCI, and NDC in the 65-74 (A), 75-84 (B), and over 85 (C) years old groups.

The results of the 75-84 years old group were compared by a one-way analysis of variance followed by the Tukey test. Due to the small number of participants aged 65-74 and over 85 years, no comparison by statistical analysis was made between the three groups.

** p<0.01.

NDC, non-demented controls; ADD, Alzheimer's disease dementia; MCI, mild cognitive impairment; MARC, mild cognitive impairment assessment tool for rapid screening using a computer.
